# Pancake-Like MOF Solid-State Electrolytes with Fast Ion Migration for High-Performance Sodium Battery

**DOI:** 10.1007/s40820-021-00628-0

**Published:** 2021-04-05

**Authors:** Gang Zhang, Jun Shu, Lin Xu, Xinyin Cai, Wenyuan Zou, Lulu Du, Song Hu, Liqiang Mai

**Affiliations:** 1 State Key Laboratory of Advanced Technology for Materials Synthesis and Processing, School of Materials Science and Engineering, Wuhan University of Technology, Wuhan, 430070 People’s Republic of China; 2 Foshan Xianhu Laboratory of the Advanced Energy Science and Technology Guangdong Laboratory, Xianhu Hydrogen Valley, Foshan, 528200 People’s Republic of China

**Keywords:** Metal–organic Frameworks, Sodium-ion Battery, Solid-like Electrolyte, Interface Contact

## Abstract

Solid-state electrolyte (SSE) of the sodium-ion battery have attracted tremendous attention in the next generation energy storage materials on account of their wide electrochemical window and thermal stability. However, the high interfacial impedance, low ion transference number and complex preparation process restrict the application of SSE. Herein, inspired by the excellent sieving function and high specific surface area of red blood cells, we obtained a solid-like electrolyte (SLE) based on the combination of the pancake-like metal–organic framework (MOF) with liquid electrolyte, possessing a high ionic conductivity of 6.60 × 10^–4^ S cm^−1^, and excellent sodium metal compatibility. In addition, we investigated the ion restriction effect of MOF’s apertures size and special functional groups, and the ion transference number increased from 0.16 to 0.33. Finally, the assembled Na_0.44_MnO_2_//SLE//Na full batteries showed no obvious capacity decrease after 160 cycles. This material design of SLE in our work is an important key to obtain fast ion migration SLE for high-performance sodium-ion batteries.

**Supplementary Information:**

The online version contains supplementary material available at 10.1007/s40820-021-00628-0.

## Highlights

- A pancake-like morphology solid-like eletrolyte of sodium battery with high ionic conductivity (6.60 × 10^–4^ S cm^−1^) was obtained by simple hydrothermal method.
- Solid-like electrolyte with pancake-like morphology showed good interface contact and excellent compatibility (stable cycle over 500 h at 0.6 mA cm^−2^) with sodium metal.
- Provides possible repulsive force explanation for the restriction of ion transport by MOF.

**Supplementary Information:**

The online version contains supplementary material available at 10.1007/s40820-021-00628-0.

## Introduction

With the development of science and technology, energy storage systems play an important role in portable equipment, transportation and electrostatic energy storage [1, 2]. As a potential energy storage system, sodium-ion batteries (SIBs) with rich sodium resources can meet most of the daily energy storage requirements [3–6]. However, traditional commercial liquid organic electrolytes in SIBs are likely to cause safety problems due to the flammability and leakage of electrolyte [7, 8]. Compared with liquid electrolyte (LE), solid-state electrolytes (SSEs) have a higher safety due to their high mechanical strength and wide voltage windows [9–12]. Nevertheless, SSEs have suffered some serious problems such as high interfacial impedance, low ion transference number and complex preparation process. Several methods were proposed to deal with the challenges of SSEs, but it is still far from the requirements of high-performance SSEs. For example, Gerbaldi et al*.* fabricated a polymer electrolyte with a single ionic conductor, increasing the ion transference number by sacrificing ionic conductivity [13]. Goodenough et al*.* improved the interface contact between the Na_3_V_2_(PO_4_)_3_ and the Na_3_Zr_2_(Si_2_PO_12_) electrolyte by adding succinitrile, but this plastic-crystalline electrolyte was not very stable to sodium metals [14]. Hence, it is crucial to exploit a kind of novel SSEs with low interfacial impedance and high ion transference number.

On the one hand, high interfacial impedance results from poor contact between SSEs and electrodes [15]. This interface gap contact limits the overall sodium ion transport. Therefore, it is more advantageous to find a kind of SSEs that can quickly transfer sodium ions in the bulk phase as liquid electrolyte. Recently, metal–organic framework (MOF) has been widely used in many fields for its high specific surface area, permanent pore, adjustable function and simple preparation process [16–20]. Numerous works have focused on the use of MOF in the field of ion battery separator and solid-like electrolyte (SLE), where MOF's own large "cage" electrical insulation can be used to store LE and sieve different ions [21–24]. For example, Wang et al*.* proposed an interface wetting effect in lithium-ion batteries by compounding MOF with ionic liquid, which significantly improved the electrolyte/electrode interfacial impedance [21]. However, it is a pity that ionic liquid is easy to react with sodium metal, and the application of MOF in sodium ion SSEs is still very scarce. Furthermore, the MOF with appropriate pore size and high specific area has more advantages in electrochemical performance [25]. On the other hand, it is well known that red blood cells allow only some of the beneficial small molecules to pass through, which will encourage the design of SLE and hopefully increase ion transference number [26, 27]. Due to their pancake-like morphology, red blood cells have a higher specific surface area that allows them to transport more electrolytes and oxygen [28, 29]. In addition, the structural stability of the pancake-like morphology of red blood cells in human body is conducive to the design of SLE with good liquid retention and high safety to a certain extent. Considering the above reasons, it is an effective strategy to select a MOF with pancake-like morphologies to optimize ion transport and interfacial impedance in sodium-ion SLE.

Herein, pancake-like MIL-125 (PLM) with shape similar to red blood cells was prepared by a simple hydrothermal method. Its unique three-dimensional pore structure can be used as a “host” for LE and restrict the migration of anions (PLM@LE). Sodium ions can be transported quickly in the PLM due to the large size, high pore ratio and smooth pancake-like morphology, making SLE exhibit a high ionic conductivity of 6.60 × 10^–4^ S cm^−1^ at room temperature and low activation energy of 0.112 eV. Meanwhile, unique aperture sizes and nucleophilic functional groups of PLM can limit the transport of anions, increasing the sodium ion transference number from 0.16 to 0.33. Moreover, the LE inside the MOF can wet the point-to-point contact of MOF particles on the atomic level. This special wetting effect and good contact between PLM particles reduces the migration barrier of sodium ion, resulting in good compatibility between PLM@LE and sodium metal. Finally, the assembled Na_0.44_MnO_2_//PLM@LE//Na SIBs can stable cycle for 160 cycles at 100 mA g^−1^ without obvious capacity degradation.

## Experimental Section

### Materials Synthesis

p-Phthalic acid (PTA 99%), 1-Methyl-2-pyrrolidinone (NMP 99.9%), 2-amino-terephthalic acid (98%) were purchased from Aladdin. Titanium isopropoxide (97%) was purchased from Macklin. *N*,*N*-Dimethylformamidel (DMF 99.5%), Methanol (99.5%), Isopropanol (99.7% IPA) were purchased from SCR. NaClO_4_-PC with 5% FEC was purchased from DuoDuo. The reagents obtained were not further purified.

Synthesis of PLM: Typically, 3.0 g PTA was added to the mixture of 54 mL DMF and 6 mL methanol, stirring continuously until the solution was transparent. Then slowly drop 1.56 mL titanium isopropoxide and transfer it into the reactor for hydrothermal reaction at 150 °C for 24 h. The obtained material was centrifugally washed with DMF and methanol three times, and placed in a 70 °C vacuum oven overnight. The dried powder was activated in the vacuum at 120 °C and stored in the argon glove box (water, oxygen < 0.1 ppm) waiting for use.

Synthesis of decanedron-like MIL-125 (DLM): 0.45 mL titanium isopropoxide and 0.747 g PTA were dissolved in the mixture of 13.5 mL DMF and 1.5 mL methanol. The subsequent hydrothermal time and temperature, as well as washing sample operation are consistent with PLM.

Synthesis of NH_2_-MIL-125: 0.45 mL titanium isopropoxide and 0.816 g 2-amino-terephthalic acid were dissolved in the mixture of 11.25 mL DMF and 3.75 mL methanol. The subsequent hydrothermal time and temperature, as well as washing sample operation are consistent with PLM.

Preparation of PLM@LE: Typically, a certain amount of activated PLM was compounded with different contents of NaClO_4_-PC with 5% FEC electrolyte, and placed at 40 °C of heating platform for 6 h.

Preparation of PLM@LE flexible membrane: PLM powder and polytetrafluoroethylene (PTFE) were ground evenly in IPA with a mass ratio of 9:1 by hand grinding. The obtained slurry was rolled into a thin film and cut into a small self-standing wafer with a thickness of about 120 μm. After that, PLM flexible film was placed in a 60 °C vacuum oven overnight to remove the excess IPA. A few drops of LE were added to the dried flexible membrane during the assembly of the battery.

Preparation of cathode: Na_0.44_MnO_2_ active material was synthesized according to the literature [30]. The working electrode was made by mixing Na_0.44_MnO_2_ (70 wt%), PLM@LE (10 wt%), acetylene black (10 wt%) and polytetrafluoroethyle (PVDF 10 wt%) in NMP to get the uniform slurry. The slurry was coated on the Al foil and dried on a 70 °C coating machine for 12 h. The electrode was cut into slices with the mass loading of 0.8–1.2 mg cm^−2^.

### Materials Characterization

Crystal structure information of sample was conducted by D8 Discover X-ray diffractometer (XRD) using Cu-Kα X-ray source with radiation. Field emission scanning electron microscopy (FESEM, JEOL-7100F) and Energy-Dispersive Spectroscopy (EDS) were used to analyze morphology and element distribution. Transmission electron microscopy (TEM) images were obtained by using JEM-2100F with acceleration voltage 200 kV. Fourier transform infrared Spectroscopy (FTIR) measurements were collected by using Nicolet 6700 (Thermo Fisher Scientific Co., USA) IR spectrometer with wavenumber range from 400 to 4000 cm^−1^. Thermogravimetric analysis (TGA) was conducted with a STA-449C from 30 to 600 °C. The Tristar II 3020 instrument was performed to measure BET specific surface area and pore volume by adsorption of nitrogen at 77 K. The X-ray photoelectron spectroscopy (XPS) analysis was tested by ESCALAB 250 Xi spectrometer. Raman spectroscopy was performed on Renishaw INVIA micro-Raman spectroscopy system.

### Electrochemical Measurements

100 mg PLM@LE was pressed into blocks at 6 T pressure and assembled into CR2025 coin cells for Electrochemical impedance spectroscopy (EIS) test. The ionic conductivity of stainless steel (SS)//PLM@LE//SS was measured from 0.1 Hz to 1000 kHz by AutoLab PGSTAT302N. The electrochemical window of SS//PLM@LE//Na was tested by linear sweep voltammetry (LSV) from 1 to 6 V at a scan rate of 1 mV s^−1^. Na//PLM@LE//Na symmetric battery was assembled and tested on the multichannel battery testing system (LAND CT2001A) to monitor Na plating/stripping test. Cathode mixture, PLM@LE flexible membrane and sodium metal anode were assembled into CR2016 coin cell for galvanostatic charge/discharge measurements and rate performance test.

## Results and Discussion

### Morphology and Structure Characterization

**Fig. 1:**
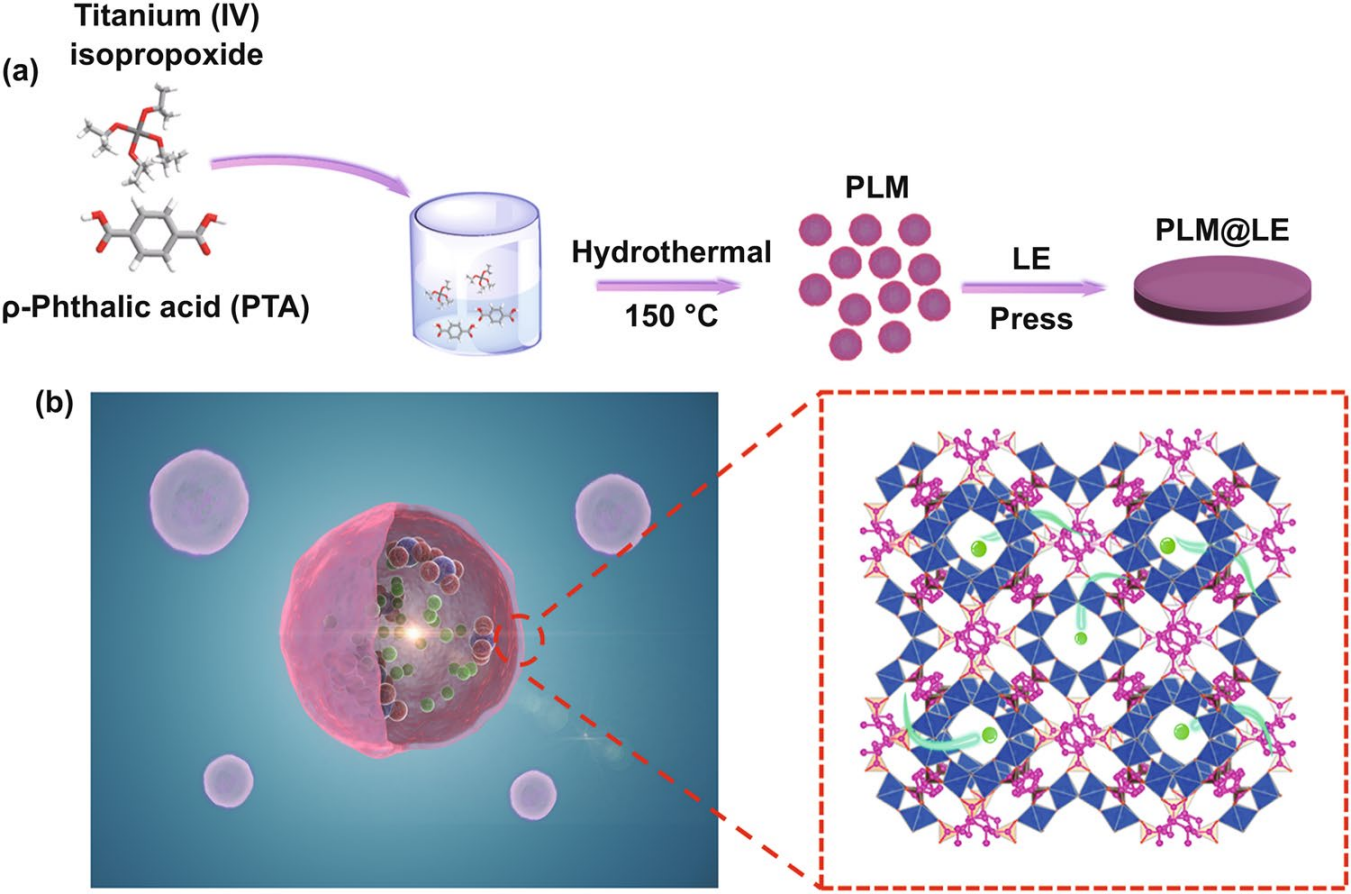
**a** Synthetic strategy of PLM@LE. **b** Top view morphology and crystal structure of PLM (green ball represents sodium ion, and the blue red ball represents other ions or molecules)

High-performance SLE with pancake-like morphology were synthesized by a simple hydrothermal method (Fig. 1a). The LE we chose is NaClO_4_-PC with 5% FEC (NaClO_4_ content is 1 M), in which PC is polycarbonate (C_4_H_6_O_3_) and FEC is fluoroethylene carbonate (C_3_H_3_FO_3_). NaClO_4_ has high decomposition temperature and high ionic conductivity, while the PC solution has good electrochemical stability and excellent ion transport capacity (FEC is used as an additive to reduce the viscosity of electrolyte) [9, 31]. As shown in Fig. S1, the longest diameter of PC and FEC molecules is 5.165 and 4.556 Å, respectively. PLM was chosen as the MOF material, which is composed of ring clusters with secondary building unit of Ti_8_O_8_ (regular octahedron in blue) and organic ligand O_2_C–C_6_H_4_–CO_2_ (magenta). It has two kinds of cages: regular tetrahedron 6.13 Å and octahedron 12.55 Å, and its apertures size are mainly distributed in 5–7 Å [32]. As shown in Fig. 1b, PLM has a pancake-like morphology, which can act as a "host" to hold the LE. In addition, its unique pore sizes can provide unrestricted transport channels for Na^+^ and limit the migration of other ions or molecules.

**Fig. 2:**
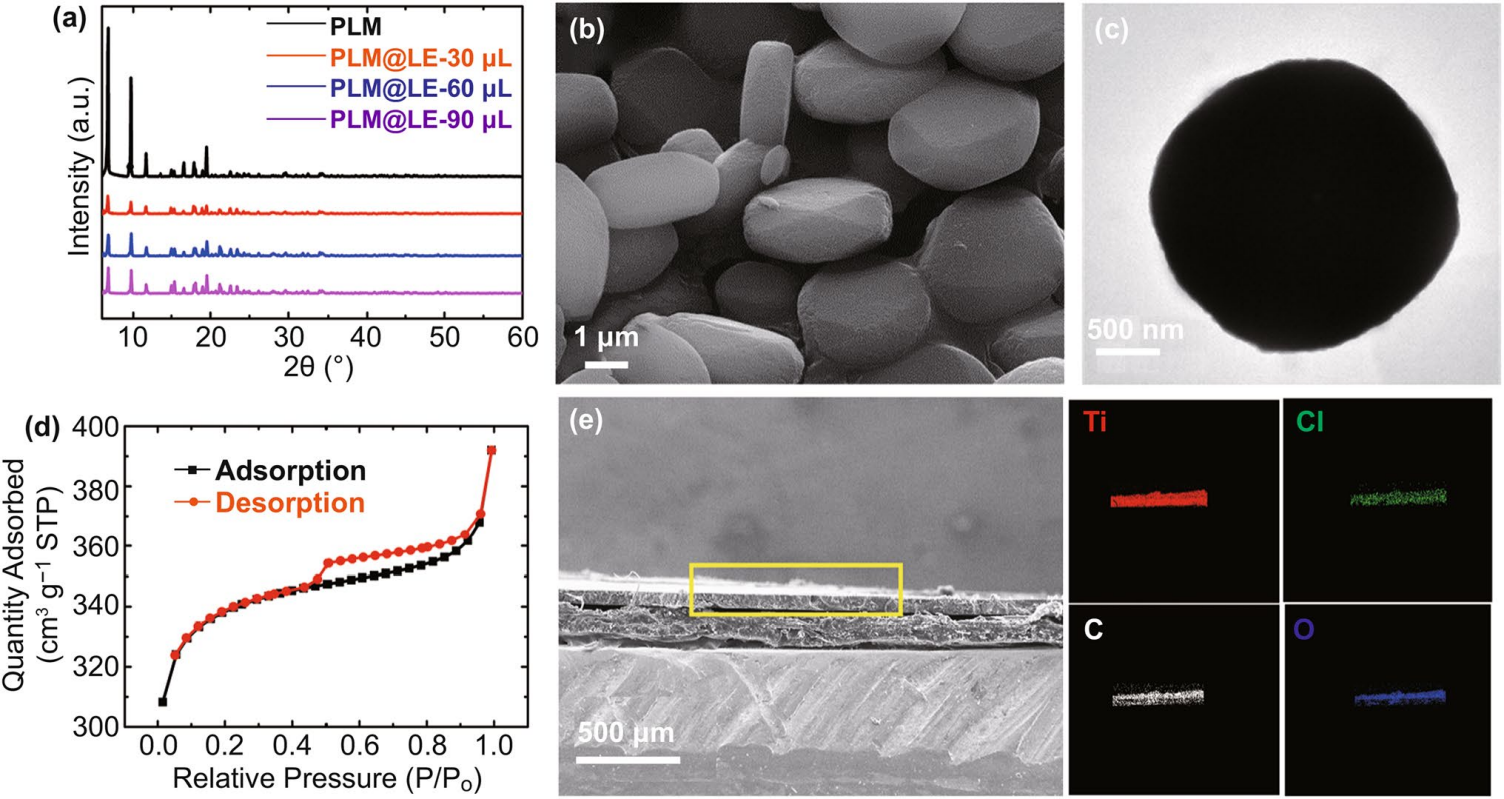
**a** XRD patterns of PLM and PLM with different LE contents. **b** SEM and **c** TEM image of PLM. **d** N_2_ adsorption/desorption isothermal linear plots of PLM. **e** Cross-sectional SEM and EDS images of PLM

X-ray diffraction (XRD) peaks of PLM at 6.7°, 9.7°, and 11.6° correspond to the (101), (200), and (211) crystallographic planes (Fig. 2a). The XRD pattern fits with the previous literature [32], indicating that PLM has been successfully synthesized. Scanning electron microscopy (SEM) and transmission electron microscopy (TEM) were used to characterize the morphology of PLM. It was obvious that PLM had a pancake-like morphology with a size of 2 μm (Fig. 2b, c). To minimize the occupation of PLM pores by other molecules, PLM powder was dried in an 120 °C vacuum oven overnight, and the fixed amount of PLM (0.1 g) was mixed with different contents of LE. The obtained PLM@LE powder was put in the glove box, waiting for the following tests. Similar to the high specific surface area of red blood cells, the BET surface area of PLM was 1112.4 m^2^ g^−1^ and the total pore volume was 0.46 cm^3^ g^−1^ according to the nitrogen adsorption/desorption isothermal tests, confirming the high porosity of PLM host (Fig. 2d). The BET surface area of PLM@LE powder decreased to 2.6 m^2^ g^−1^ indicates that the LE has successfully occupied the channel of PLM (Fig. S2). With the successful implantation of different LE contents, the PLM skeleton did not appear obvious collapse which was proved by XRD patterns (Fig. 2a) and Raman spectra (Fig. S3) of PLM@LE. Although there was no characteristic peak of LE, the intensity of the PLM@LE peaks decreased obviously, which indicated that LE existed in the internal framework of PLM. The SEM images of PLM with different contents LE were shown in Fig. S4. With the increase in LE content, the pancake-like morphology of PLM has no obvious change. In addition, PLM@LE samples with different LE were further analyzed by X photoelectron spectroscopy (XPS) as shown in Fig. S5. It is worth mentioning that the excessive LE (120 μL) was difficult to detect by XPS. A strong Cl peak was detected with the addition of LE, and the C peak based on PLM had no position shift, which indicates that there was no change of valence electrons with the mix of LE.

In order to explore the advantages of the pancake-like morphology of PLM, a decahedron-like material about 2 µm was prepared with different solvent contents, as shown in Fig. S6a, b. XRD peaks at 6.7°, 9.7°, and 11.6° fully demonstrate that the decanedron-like MOF (DLM) was still MIL-125 (Fig. S6c). The BET surface area and pore volume of DLM was 1049.9 m^2^ g^−1^ and 0.43 cm^3^ g^−1^, respectively (Fig. S6d). Compared with PLM, the internal pore diameter distribution of DLM was almost the same as PLM, which indicates the consistency of the internal aperture distribution between PLM and DLM (Fig. S6e, f). Furthermore, the specific surface area of DLM decreased, which was caused by the decrease in external specific surface area or/and missing linkers. To study the influence of structural defects or site impurities of MOF on the electrochemical performance, we added the NH_2_ functional group to the PLM (NH_2_-MIL-125). It is obvious that NH_2_-MIL-125 still has a pancake-like morphology, and the inset shows that the NH_2_-MIL-125 is a yellow powder (Fig. S7a). In addition, the XRD peaks maintains a good similar crystal pattern of PLM (Fig. S7b). FTIR spectrum was used to further analyze the structure of NH_2_-MIL-125, and it was found that there were two small peaks at 3467 and 3436 cm^−1^, which represented the contraction vibration peaks of NH_2_ (Fig. S7c). Subsequently, the high specific surface area of NH_2_-MIL-125 (1126.9 m^2^ g^−1^) was found, indicating that the NH_2_ had no significant effect on the specific surface area (Fig. S7d).

At the same time, the thermal stability of PLM, PLM@LE and DLM was tested (Fig. S8). There are two stages of weight loss in PLM. In the first stage, the weight loss is in the range of 80–120 °C, mainly cause by the loss of water or solvent molecules in the pores. In the second stage, the weight loss begins at about 300 °C and ends at about 450 °C. This is due to the collapse of the framework accompanied by the decomposition of organic ligands [32]. The thermal stability of DLM was similar to PLM, indicating that the change of crystallographic planes proportion has little influence on the thermal stability. A sharp decline of PLM@LE occurred between 100 and 200 °C due to the addition of LE, but the subsequent decomposition trend of high temperature section was same as the PLM skeleton, indicating that LE was almost completely decomposed at 200 °C. Moreover, PLM@LE white powder was pressed under 6 T pressure and kept the pressure for one minute. The PLM was closely packed together with no obvious cracks on the surface (Fig. S9a, b). High structural stability similar to red blood cells, pancake-like morphology reduced the stress on a single PLM, ensuring the high safety of PLM@LE under high pressure. However, owing to the decahedron-like morphology of DLM, the structure of DLM collapsed and broke obviously under the same pressure, leading to the increase in sodium ion transport barrier in DLM (Fig. S9c, d). The cross-sectional SEM and the energy-dispersive spectrometer (EDS) elemental mappings show that the overall thickness of the SLE was about 120 μm and the Cl element was uniformly distributed in the PLM@LE, which implies that the LE remains in the PLM after pressed at 6 T high pressure (Fig. 2e).

### Electrochemical Performance and Ion Transport Mechanism

**Fig. 3:**
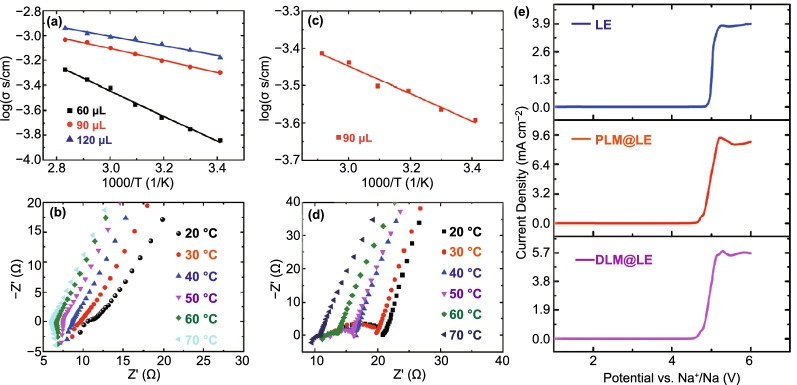
**a** Arrhenius plots for the ionic conductivity of PLM@LE with different LE contents. **b** EIS of PLM@LE (0.1 g: 90 μL) at temperatures from 20 to 70 °C. **c** Arrhenius plot and **d** EIS of DLM@LE (0.1 g: 90 μL) at different temperatures. **e** LSV of DLM@LE, LE and PLM@LE under a scan rate of 1 mV s^−1^ at 23 °C

The content of LE is directly related to the electrochemical properties of PLM@LE. The PLM@LE with different LE were assembled into stainless steel (SS)//PLM@LE//SS to perform electrochemical impedance spectroscopy (EIS) at 20–70 °C. The Arrhenius curves with different LE contents were shown in Fig. 3a. With the LE content increase from 60, 90 to 120 μL, the activation energy of PLM@LE was 0.199, 0.112, and 0.082 eV, respectively. The activation energy indicates the Na^+^ migration ability in PLM organic skeleton. The lower the activation energy value, the easier the Na^+^ transport. But unfortunately, 120 μL PLM@LE was no longer a dry-solid powder, which represents that it cannot completely absorb LE (Video S1). Therefore, the optimal ratio (MOF: LE = 0.1 g: 90 μL) was determined and used for the subsequent testing. The EIS curves of different temperatures were shown in Fig. 3b. The ionic conductivity of PLM@LE at 20 and 70 °C was 6.60 × 10^–4^ and 1.15 × 10^–3^ S cm^−1^, respectively. In addition, DLM was compounded with 90 μL of LE and assembled into SS//DLM@LE//SS for the same Arrhenius and EIS tests. The activation energy of DLM@LE was 0.135 eV, which was higher than 0.112 eV of PLM@LE (Fig. 3c, d). The ionic conductivity of DLM@LE was 2.56 × 10^–4^ and 3.86 × 10^–4^ S cm^−1^ at 20 and 70 °C, respectively. The difference between PLM@LE and DLM@LE was ascribed to the high liquid retention of pancake-like morphology. What' more, the ionic conductivity at room temperature and activation energy of NH_2_-MIL-125 was 6.08 × 10^–4^ S cm^−1^ and 0.1167 eV, respectively (Fig. S10). These results indicate that the electrochemical properties of the pancake-like MOF after ammoniation are still very similar to PLM@LE, although the internal sites of MOF are changed. Electrochemical windows of PLM@LE and DLM@LE were measured at room temperature by linear sweep voltammetry (LSV), as shown in Fig. 3e. The electrochemical windows of PLM@LE and DLM@LE were stable in the range of 1–4.8 V, indicating the electrochemical stability of MOF. To further demonstrate the electrochemical stability of MOF, CV tests were performed at voltages from − 0.5 to 2 V. It can be clearly seen that CV curves in PLM@LE can maintain a high degree of consistency after CV cycles, indicating that MOF has a good stability to sodium metal (Fig. S11).

**Fig. 4:**
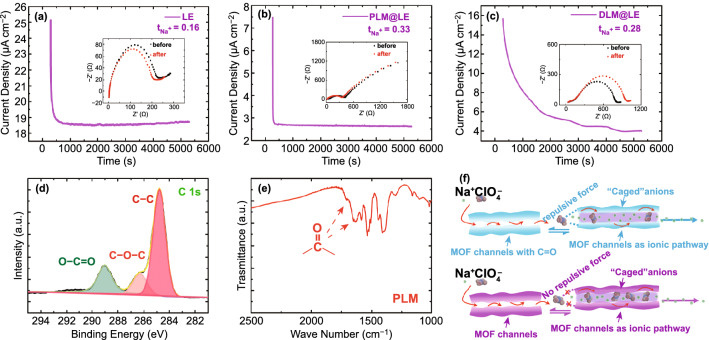
Current–time profile for **a** Na//LE//Na, **b** Na//PLM@LE//Na and **c** Na//DLM@LE//Na cell. Inset: EIS before and after polarization. **d** High-resolution XPS spectrum of C 1 s for PLM. **e** FTIR spectrum of PLM. **f** Schematic of the selective Na^+^ ionic transport imposed by the MOF host

The sodium ion transference number (*t*_Na+_) was tested by assembling a Na symmetrical cell at room temperature with a constant voltage of 10 mV according to the literature method (Fig. 4a–c) [33]. According to the EIS test, the interface resistance of PLM@LE against sodium was lower than DLM@LE, which matched the previous EIS test. In addition, the *t*_Na+_ of the initial LE was only 0.16, indicating the main type of ionic conductance was ClO^−^_4_ rather than Na^+^. The *t*_Na+_ of PLM@LE and DLM@LE increased significantly to 0.33 and 0.28 because of the aperture size of MIL-125 host. The size of its pore is about 5 Å, which could restrict migration of PC (5.165 Å) and FEC (4.556 Å). According to the previous report, PC molecules and Na^+^ tend to interact with each other near carbonyl oxygen atoms, which increases the diffusion barrier of Na^+^ [34]. More recently, Chang et al*.* have realized the de-solvated of a lithium-ion electrolyte through an ordered hole in the electrically insulated MOF [35]. Therefore, limiting some of the solvent molecules migration will increase the de-solvated process of Na^+^ and the *t*_Na+_. What’s more, we provide another possible explanation, because the strong electronegativity of some special functional group leads to an increase in the density of the electron cloud, and there might be a repulsive force against the electron-rich material [36, 37]. Therefore, XPS, FTIR and Raman spectroscopy were used to analyze the existence of special functional groups on the MOF skeleton. High-resolution XPS spectrum of C 1 s for PLM in Fig. 4d showed that the three peaks at binding energies of 288.67, 286.27, and 284.77 eV corresponded to covalent O–C = O, C–O–C, and C–C bonds, [38]. In addition, the high-resolution XPS spectra of O 1 s reflected the existence of covalent C=O bond at the binding energy of 532.22 eV (Fig. S5) [39]. Moreover, FTIR spectrum further proved the existence of C=O. As shown in Fig. 4e, the FTIR peaks at 1706 and 1653 cm^−1^ represent the stretching vibration of carbonyl function C=O [40]. The strong electronegativity of oxygen in the carbonyl group makes carbonyl have a strong nucleophilic property. Strong repulsive force could restrict the migration of anions, which leads to the increase in *t*_Na+_ (the mechanism diagram was shown in Fig. 4f). DLM was characterized by FTIR, as shown in Fig. S12a. C=O peaks appeared at 1715 and 1628 cm^−1^, and the overall peak positions corresponded to PLM, indicating the consistency of organic functional groups. Raman spectra in Fig. S12b further demonstrate the existence of C=O and the consistency between PLM and DLM. The four strong peaks at 1618, 1435, 1146, and 865 cm^−1^ correspond to the bending vibrations and stretching of the C=O and the C=C, C–H vibrations on the benzene ring.

### Interface Analysis and Solid-State Battery Performance

**Fig. 5:**
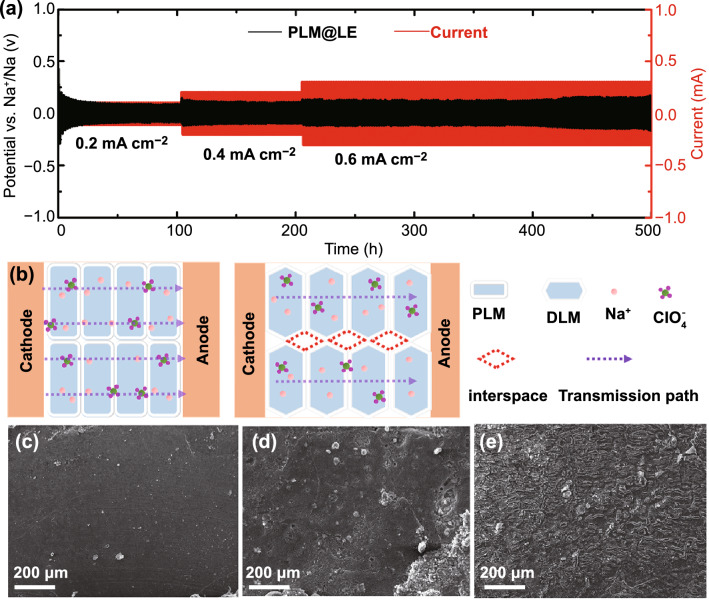
**a** Direct current Na plating/stripping of Na//PLM@LE//Na at current densities of 0.2, 0.4 and 0.6 mA cm^−2^. **b** Schematic of the PLM@LE and DLM@LE internal interface contact. SEM images of sodium metal **c** original, **d** Na//PLM@LE//Na and **e** Na//DLM@LE//Na

The direct current (DC) Na plating/stripping experiments were used to measure the impedance of sodium ion migration at the interface and the stability of SLE against sodium metal. Figure 5a shows the voltage–time plots of the Na//PLM@LE//Na symmetric cells at different current densities. The polarization voltage of PLM@LE was no more than ± 90, ± 120, and ± 150 mV at 0.2, 0.4, and 0.6 mA cm^−2^, respectively. This low interfacial impedance and excellent interfacial compatibility was ascribed to the wetting effect of LE on the interfaces. The contact between MOF and sodium metal was not completely solid–solid contact, which constructed a fast path for the rapid transport of sodium ions in interfaces [41]. In addition, PLM@LE can cycle steadily for more than 500 h, representing the interfacial stability between the PLM@LE and sodium metal during the cycle. In addition, the three characteristic peaks of PLM still exist obviously at 6.7°, 9.7°, and 11.6°, indicating that PLM has good chemical stability after the cycling process (Fig. S13). The voltage–time plots of the PLM@LE and DLM@LE at 0.4 mA cm^−2^ current density were shown in Fig. S14. It can be seen that the polarization of DLM@LE increases significantly during the cycle, which indicates the poor compatibility with sodium metal. The reason for this phenomenon was shown in Fig. 5b. From the microscopic point of view, the good contact between the smooth PLM leads to a lower sodium ion transport barrier in PLM interfaces. In addition, sodium dendrites tend to grow in the cracks of DLM@LE during the cycling process, resulting in the fragmentation or pulverization of electrolytes. The SEM image of sodium metal before cycling was shown in Fig. 5c, which shows that the surface of sodium metal was very smooth without the appearance of sodium dendrites. Compared with DLM@LE, the dendrite growth of sodium metal on PLM@LE was inhibited to a greater extent because the sodium dendrite did not grow conveniently in the PLM interstitial space (Fig. 5d, e). Figure S15 represents the inhibition of sodium dendrite in PLM@LE at different current densities, which indicates the electrochemical superiority of PLM@LE.

**Fig. 6:**
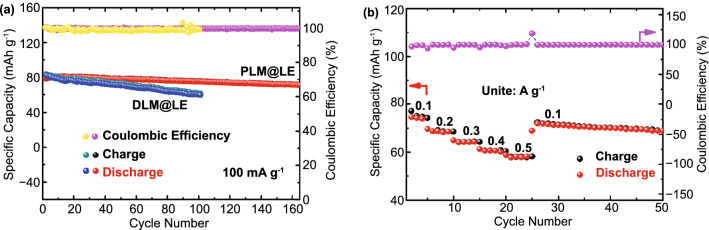
**a** Cycling performance of Na_0.44_MnO_2_//PLM@LE//Na and Na_0.44_MnO_2_//DLM@LE//Na solid batteries at 100 mA g^−1^. **b** Rate performance of Na_0.44_MnO_2_//PLM@LE//Na

To further explore the application prospect of PLM@LE, Na_0.44_MnO_2_//PLM@LE//Na full battery was assembled. The charge/discharge cycling at 100 mA g^−1^ current density was shown in Fig. 6a. It is worth mentioning that the capacity has a standard deviation of 4 mAh g^−1^. The discharge capacity has a slight increase from 79.4 to 80.9 mAh g^−1^ in the first 12 cycles. This is because the interface will gradually be completely infiltrated by the LE during the cycling process, leading to an increase in capacity, which was consistent with the DC test results. After 160 cycles, the specific capacity decreased slightly to 71.4 mAh g^−1^. At the same time, the full battery of DLM@LE showed an initial specific capacity of 83.1 mAh g^−1^ at a current density of 100 mA g^−1^, but the capacity continued to decline after 100 cycles with a capacity retention rate of 72.9%. Subsequently, rate performance of PLM@LE was measured at different current densities of 100, 200, 300, 400, 500, and 100 mA g^−1^, and the discharge capacities were 74.3, 68.8, 64.3, 60.6, 58.1, and 72 mAh g^−1^ (Fig. 6b), which fully demonstrated that PLM@LE has a high structural stability and a good recoverable cycle. What’s more, the cross-sectional SEM image of Na_0.44_MnO_2_//PLM@LE (Fig. S16) and EIS tests before and after the cycling (Fig. S17) can provide further evidence of good contact between PLM@LE and the electrode. The findings showed that PLM@LE possesses high ionic conductivity, excellent interfacial compatibility and simple preparation technology. All the above results indicate that PLM@LE has a high application value in high-performance SIBs.

## Conclusion

In summary, we constructed a novel SLE based on a MOF with pancake-like morphology. This special morphology of MOF makes PLM have a higher specific surface area to accommodate more LE and block the transport of anions, so that PLM@LE has a high ionic conductivity (6.60 × 10^–4^ S cm^−1^) and higher sodium ion transference number (0.33). In addition, by analyzing the influence of different morphology of SLE, it was found that PLM@LE had better interface contact, which inhibited the growth of sodium dendrite to a great extent and shown good sodium metal compatibility over 500 h. What’s more, characterization of C=O functional groups on MOF provides another possible repulsive force explanation for the restriction of anion transport. Finally, after 160 cycles at 100 mA g^−1^, the specific capacity retention rate of the assembled full battery was 88%. This work provides a novel idea for the design of fast ion transfer, high-performance Na^+^ SLE.

## Supplementary Information

Below is the link to the electronic supplementary material.

Supplementary file1 (PDF 1232 KB)

Supplementary file2 (MP4 5567 KB)
