# Supplementary material for: Pancake-Like MOF Solid-State Electrolytes with Fast Ion Migration for High-Performance Sodium Battery
Source: Nanomicro Lett. 2021 Apr 5;13:105. doi: 10.1007/s40820-021-00628-0 (PMC8021678; doi:10.1007/s40820-021-00628-0)
Supplement: Supplementary file 1 — Supplementary file1 (PDF 1232 KB) [file 40820_2021_628_MOESM1_ESM.pdf]

Supporting Information for

Pancake-Like MOF Solid-State Electrolytes with Fast Ion Migration for High-Performance Sodium Battery

Gang Zhang[1], Jun Shu[1], Lin Xu[1, 2, *], Xinyin Cai[1], Wenyuan Zou[1], Lulu Du[1], Song Hu[1], Liqiang Mai[1, 2, *]

[1]State Key Laboratory of Advanced Technology for Materials Synthesis and Processing, School of Materials Science and Engineering, Wuhan University of Technology, Wuhan 430070, People's Republic of China

[2]Foshan Xianhu Laboratory of the Advanced Energy Science and Technology Guangdong Laboratory, Xianhu Hydrogen Valley, Foshan 528200, People's Republic of China

*Corresponding authors. E-mail: linxu@whut.edu.cn (Lin Xu); mlq518@whut.edu.cn (Liqiang Mai)

Supplementary Tables and Figures

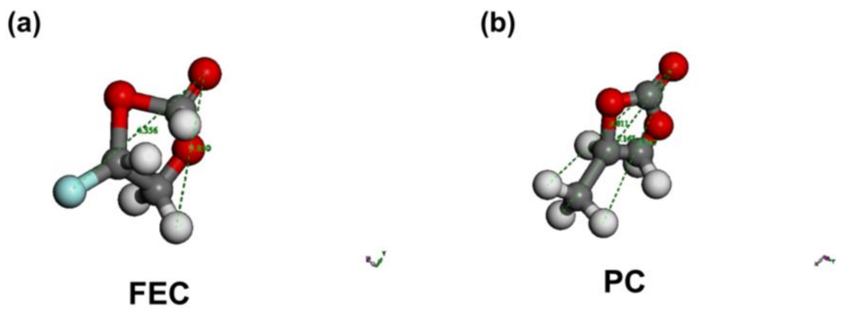

Fig. S1 Molecular model of **a** FEC and **b** PC

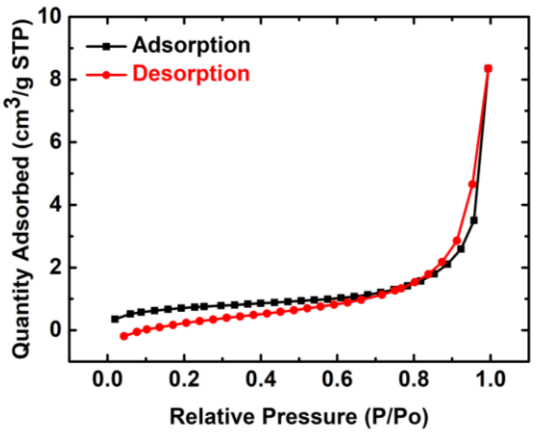

Fig. S2 N_2 adsorption/desorption isothermal linear plots of PLM@LE (0.1 g PLM : 90 μL LE)

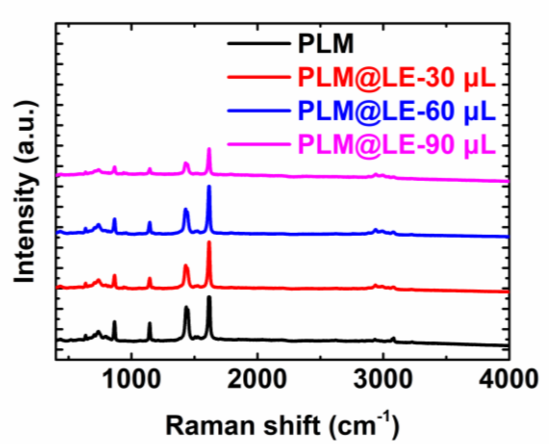

Fig. S3 Raman spectra of PLM and PLM with different LE contents

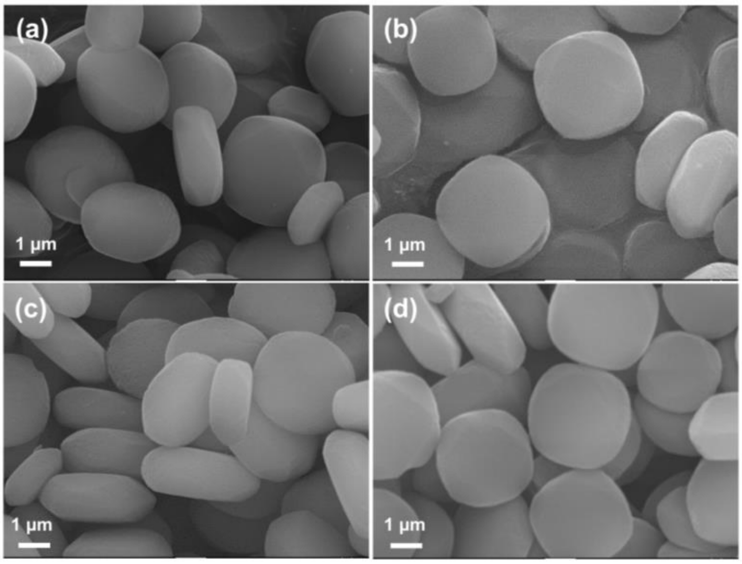

Fig. S4 SEM images of 0.1 g PLM with different contents of LE. **a** 0 μL, **b** 60 μL, **c** 90 μL and **d** 120 μL

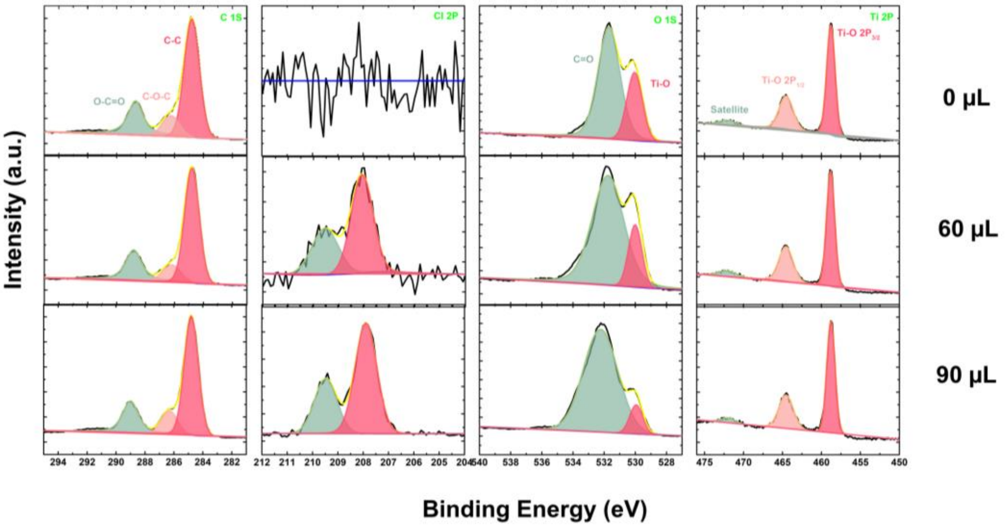

Fig. S5 XPS spectra of 0.1 g PLM with different contents of LE

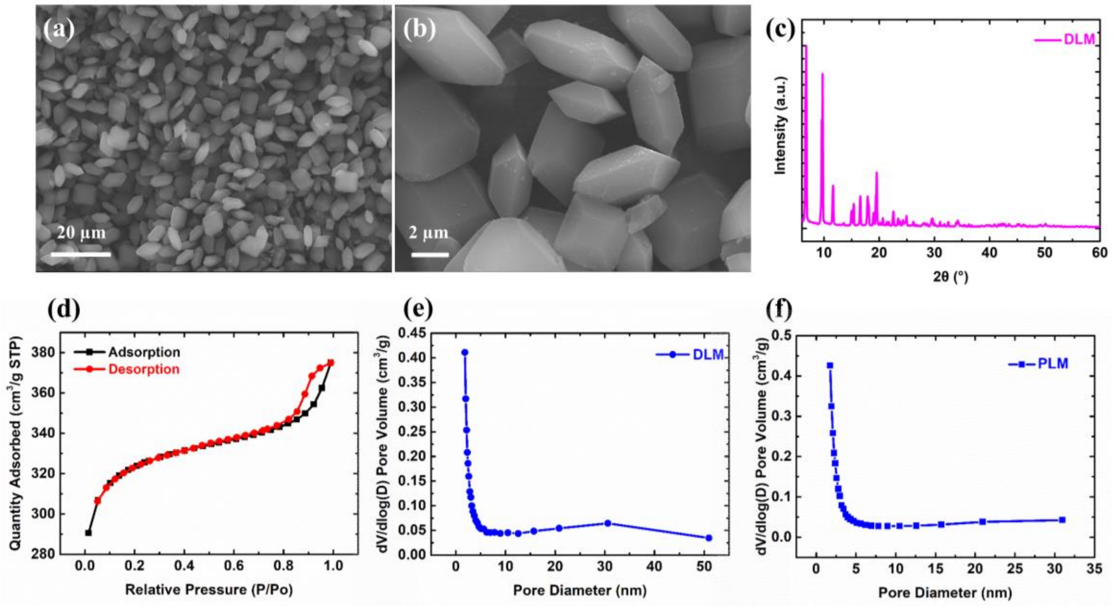

Fig. S6 a Low magnification **b** High magnification SEM images of DLM. **c** XRD pattern of DLM. **d** N_2 adsorption/desorption isothermal linear plots of DLM. Pore size distribution curves of **e** DLM and **f** PLM

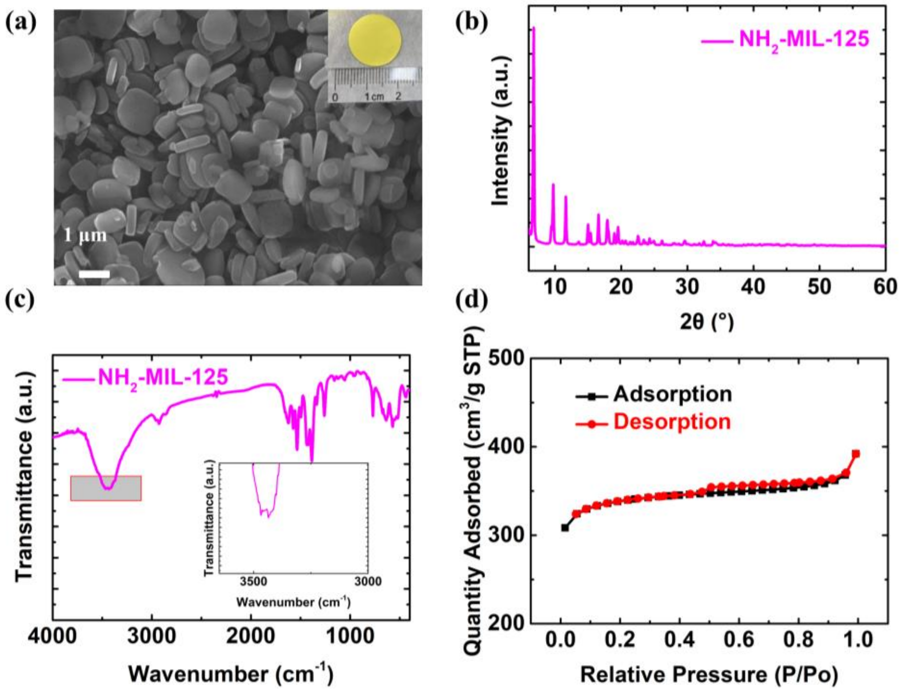

Fig. S7 a SEM image of NH_2-MIL-125. Inset: the optical image of NH_2-MIL-125 pellet. **b** XRD pattern, **c** FTIR spectrum and **d** BET plots of NH_2-MIL-125

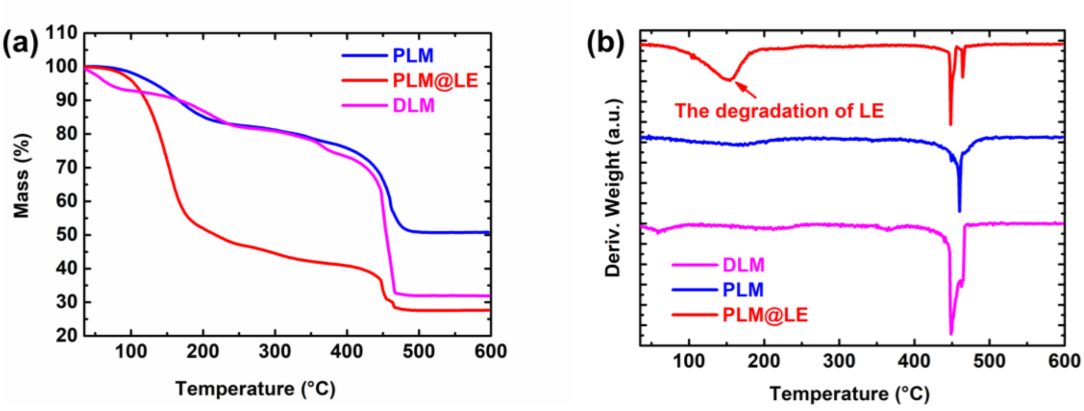

Fig. S8 a TGA and **b** DTG of PLM, PLM@LE and DLM at air atmosphere at a speed of 10 °C min^{-1}

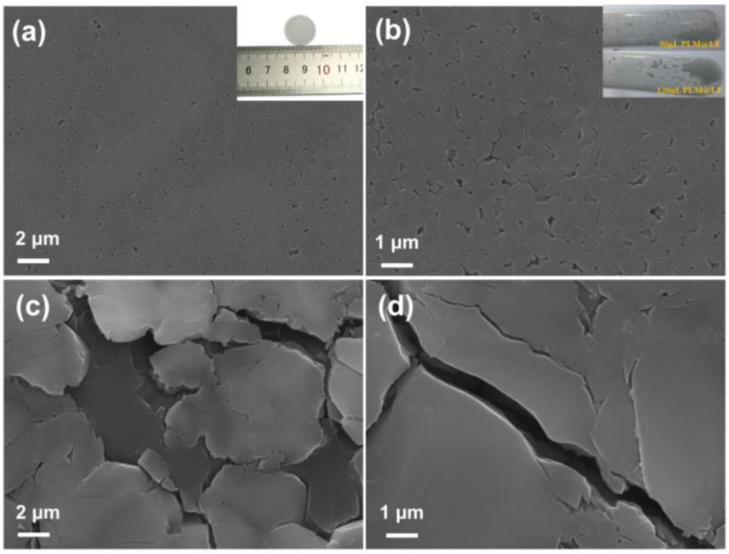

Fig. S9 a Low magnification SEM image of PLM@LE after pressing at 6 T pressure. Inset: the optical image of PLM@LE. **b** High magnification SEM image of PLM@LE. Inset: the optical image of PLM with different contents of LE. **c** Low magnification and **d** High magnification SEM images of DLM@LE after pressing at 6 T pressure

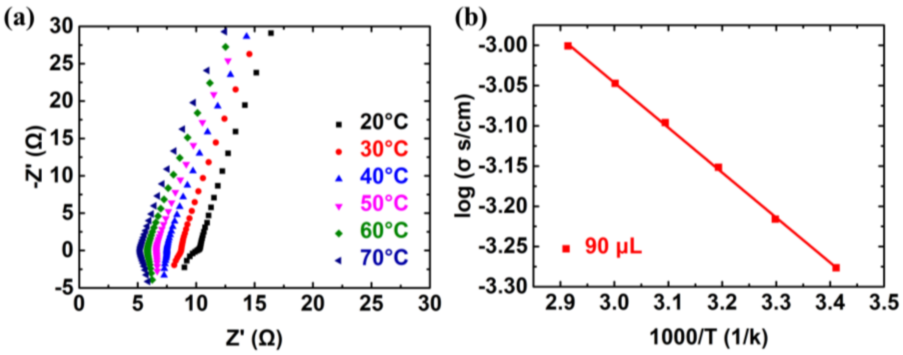

Fig. S10 a EIS at different temperature and **b** Arrhenius plot for the ionic conductivity of NH_2-MIL-125 with 90 μL LE

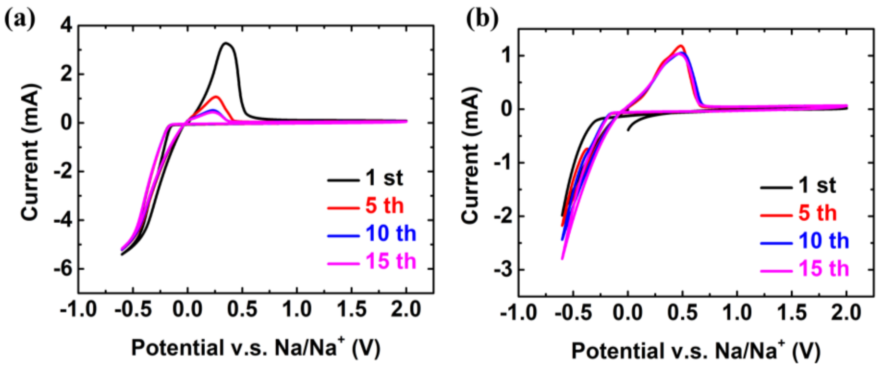

Fig. S11 CV curves of **a** LE and **b** PLM@LE under 10 mV/s scan rate

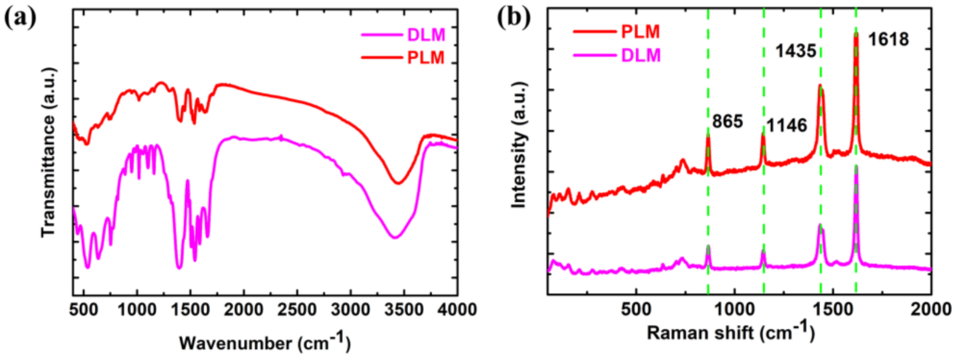

Fig. S12 a FTIR spectra and **b** Raman spectra of PLM and DLM

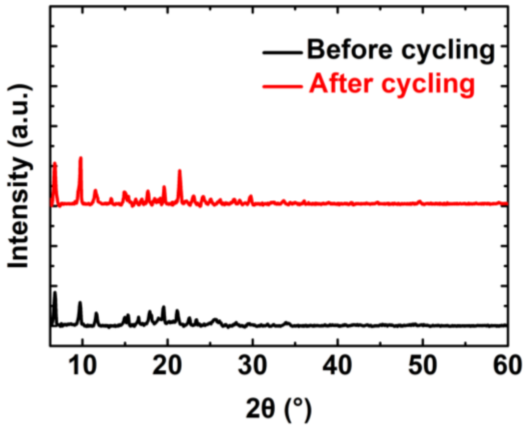

Fig. S13 XRD patterns of PLM@LE before and after the DC sodium plating/stripping cycle

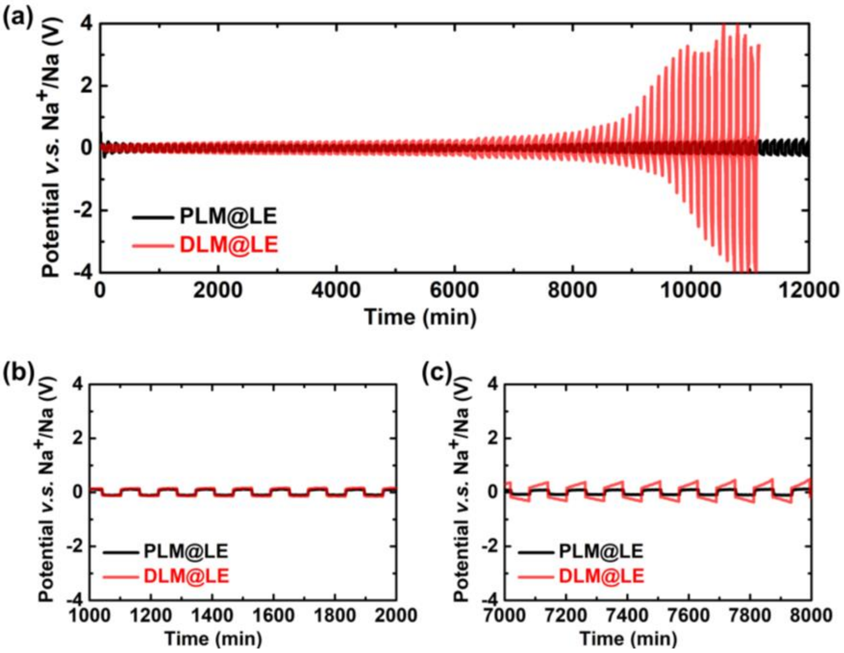

Fig. S14 a Direct current Na plating/stripping of Na//PLM@LE//Na and Na//DLM@LE//Na cell. Magnification images of **b** 1000-2000 min and **c** 7000-8000 min

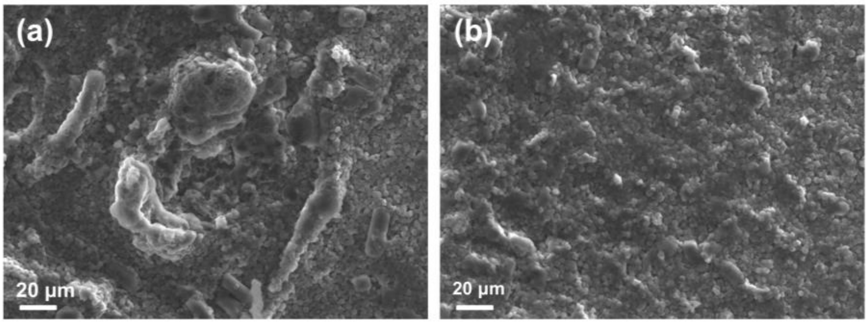

Fig. S15 SEM images of PLM@LE at different current densities after cycling. **a** 1 mA cm^{-2} and **b** 0.4 mA cm^{-2}

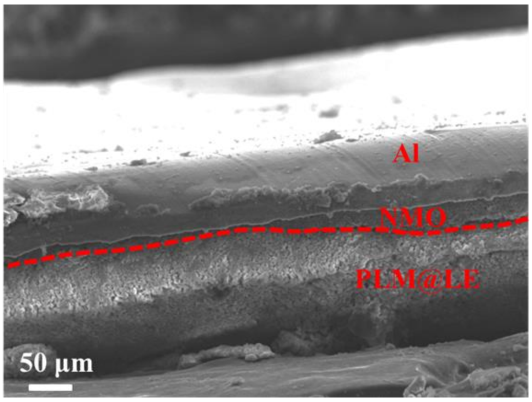

Fig. S16 Cross-sectional SEM image of $Na_{0.44}MnO_2$//PLM@LE after cycling

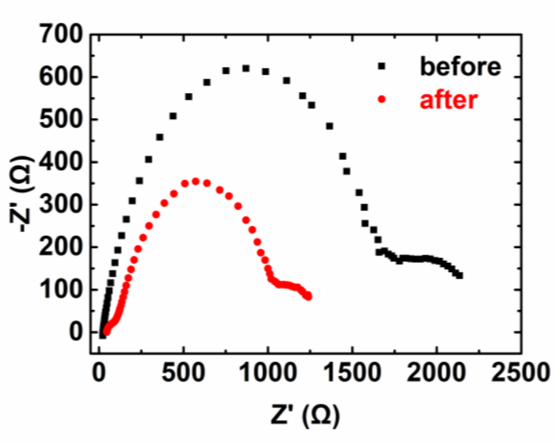

Fig. S17 EIS of PLM@LE before and after 200 cycles
